# DoMYB5 and DobHLH24, Transcription Factors Involved in Regulating Anthocyanin Accumulation in *Dendrobium officinale*

**DOI:** 10.3390/ijms24087552

**Published:** 2023-04-20

**Authors:** Kun Yang, Yibin Hou, Mei Wu, Qiuyu Pan, Yilong Xie, Yusen Zhang, Fenghang Sun, Zhizhong Zhang, Jinghua Wu

**Affiliations:** College of Horticulture, Fujian Agriculture and Forestry University, Fuzhou 350002, China; akunyang@126.com (K.Y.); wm985@outlook.com (M.W.); panqiuyu2023@163.com (Q.P.); m17805957656@163.com (Y.Z.); sunfenghang1998@163.com (F.S.)

**Keywords:** activator, anthocyanin, bHLH, *Dendrobium officinale*, MYB

## Abstract

As a kind of orchid plant with both medicinal and ornamental value, *Dendrobium officinale* has garnered increasing research attention in recent years. The MYB and bHLH transcription factors play important roles in the synthesis and accumulation of anthocyanin. However, how MYB and bHLH transcription factors work in the synthesis and accumulation of anthocyanin in *D. officinale* is still unclear. In this study, we cloned and characterized one MYB and one bHLH transcription factor, namely, *D. officinale* MYB5 (DoMYB5) and *D. officinaleb* bHLH24 (DobHLH24), respectively. Their expression levels were positively correlated with the anthocyanin content in the flowers, stems, and leaves of *D. officinale* varieties with different colors. The transient expression of *DoMYB5* and *DobHLH24* in *D. officinale* leaf and their stable expression in tobacco significantly promoted the accumulation of anthocyanin. Both DoMYB5 and DobHLH24 could directly bind to the promoters of *D. officinale CHS* (*DoCHS*) and *D. officinale DFR* (*DoDFR*) and regulate *DoCHS* and *DoDFR* expression. The co-transformation of the two transcription factors significantly enhanced the expression levels of *DoCHS* and *DoDFR*. DoMYB5 and DobHLH24 may enhance the regulatory effect by forming heterodimers. Drawing on the results of our experiments, we propose that DobHLH24 may function as a regulatory partner by interacting directly with DoMYB5 to stimulate anthocyanin accumulation in *D. officinale*.

## 1. Introduction

*Dendrobium officinale Kimura* et Migo (*D. officinale*) is a perennial herb of *Dendrobium* in Orchidaceae. With beautiful flowers and high medicinal value, it is an important ornamental and medicinal plant with a long history of artificial cultivation. Different varieties of *D. officinale* have different biological characteristics in color, plant height, and stem diameter, among which color characteristics are particularly important. The application value of different color types varies significantly [1]. For example, the red *D. officinale* perfectly integrates the needs of people for ornamental gardening and medicinal plants and has high research and utilization value.

Plant pigments mainly include chlorophyll, carotenoids, flavonoids, and alkaloids, which are responsible for different colors. Flavonoids are widely distributed in plants, giving them a wide range of colors, from light yellow to blue-purple [2,3]. Flavonoid compounds are important secondary metabolites that include anthocyanins, flavonoids, isoflavones, flavonols, and proanthocyanidins [4]. More than 600 types of anthocyanins have been identified in plants thus far [5]. There are generally six anthocyanin pigments in plants: delphinidin (Dp), peonidin (Pn), cyanidin (Cy), pelargonidin (Pg), malvidin (Mv), and petunidin (Pt) [6]. Anthocyanins play an important role in protecting plants from ultraviolet radiation, preventing pathogen infection, attracting insect pollination, and enhancing plant tolerance to low temperature, drought, high salt, and other stresses [7,8,9]. Anthocyanins are widely involved in anticancer, antioxidant, and antiviral processes, and can be used to prevent and treat cardiovascular diseases, retinal diseases, and diabetes [4,10,11,12].

The gene expression of anthocyanin synthesis in many plants is regulated by R2R3-MYB, bHLH, or MYB-bHLH-WD40 (MBW) complexes [13,14,15,16]. In different plants, MYB transcription factors have significantly different mechanisms in regulating the biological metabolism of flavonoids [17]. The MYB of monocots can regulate the biological metabolism of flavonoids by binding DNA alone or by binding bHLH into a complex [18,19,20]. bHLH plays an important role in the biosynthesis and accumulation of flavonoids in plants by binding DNA alone or with MYB as a complex [21,22,23]. The *FhMYB5* can slightly upregulate the late biosynthesis genes (LBGs) of the anthocyanin pathway, while when FhMYB5 acts with FhTT8L and FhGL3L, both early biosynthesis genes (EBGs) and LBGs of anthocyanin pathways are significantly activated [24]. A similar situation has been found in the relevant studies of lily; LhMYB12 can bind to the *LhCHS* and *LhDFR* promoters alone and can also interact with LhbHLH2 to regulate the expression of *LhCHS* and *LhDFR* [18]. Some MYB [25,26,27,28,29] and bHLH transcription factors [30,31,32,33,34] of orchids have been isolated. In *phalaenopsis*, PsUMYB6, a flower color-related R2R3-MYB family member, can activate the expression of *PsDFR*, a late gene of the anthocyanin pathway, only in the presence of bHLH protein [26]. PeMYB11 needs the assistance of PebHLH1, while PeMYB2 and PeMYB12 can activate the expression of *PeF3H5*, *PeDFR1*, and *PeANS3* with or without PebHLH1 [27].

*D. officinale* has three different color types, namely, green, red, and purple. There are few studies on the mechanism of flavonoid metabolism in *D. officinale.* The anthocyanin synthesis gene of *D. officinale* may be regulated by *DoANS* and *DoUFGT* [34]. DhbHLH1 in *D. officinale* hybrids is closely related to the synthesis of lip anthocyanins, and the co-expression of *DhMYB2* or *DhbHLH1* on white petals can directly activate the transcription of *DhF3H*, *DhDFR,* and *DhANS*, and generate purple spots on white petals [28]. DcTT8, a bHLH transcription factor of *D. officinale*, also plays an important role in anthocyanin biosynthesis. It can directly bind to the promoters of *DcF3′H* and *DcUFGT*, thereby regulating the expression of *DcF3′H* and *DcUFGT* [32]. The function and mechanism of MYB and bHLH transcription factors in the anthocyanin metabolism of *D. officinale* are still unclear. In most plants, MYB promotes anthocyanin synthesis by enhancing the expression level of LBGs [35,36,37,38]. Compared with green varieties, red *D. officinale* varieties have significantly higher expression levels of EBGs, such as *CHS* [32]. Is MYB of *D. officinale* involved in the regulation of *CHS* gene expression? Does it regulate EBGs (such as *CHS*) and LBGs (such as *DFR*) at the same time? The answers to the above questions are still unknown.

In this paper, we identified one R2R3-MYB transcription factor, DoMYB5, and one bHLH transcription factor, DobHLH24, and analyzed their functions in the anthocyanin biosynthesis of *D.officinale.* Our results indicated that *DoMYB5* in *D. officinale* can simultaneously enhance the expression levels of *DoCHS* and *DoDFR* in anthocyanin synthesis, either alone or in combination with DobHLH24. This study enriches our understanding of the mechanism of MYB regulating anthocyanin synthesis in monocots, and it also provides new ideas for cultivating high anthocyanin in *D. officinale* varieties.

## 2. Results

### 2.1. The Expression Level of DoMYB5 and DobHLH24 Was Correlated with Anthocyanin Biosynthesis

**Figure 1:**
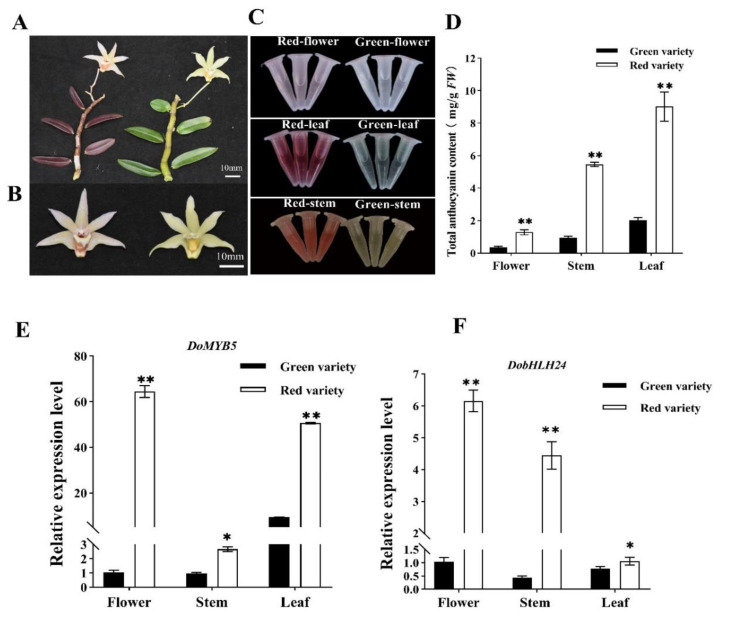
Anthocyanin content and relative expression level of *DoMYB5* and *DhbHLH24* in flower, stem, and leaf of red and green *D. officinale* varieties. (**A**,**B**) Photographs of red and green *D. officinale* varieties. (**C**,**D**) Anthocyanin extracts from the flower, stem, and leaf of red and green *D. officinale* varieties. (**E**,**F**) *DoMYB5* and *DhbHLH24* expression levels in the flower, stem, and leaf of red and green *D. officinale* varieties were measured using qRT-PCR. * Significant difference (*p* < 0.05). ** Extremely significant difference (*p* < 0.01).

After analyzing the anthocyanin content in the leaves, stems, and petals of red and green *D. officinale* varieties, it was found that the anthocyanin content of different color varieties was significantly different (Figure 1A,B,D). The anthocyanin content of the red variety was significantly higher than that of the green variety, regardless of organs (Figure 1A–D). The anthocyanin content in different parts of the red varieties was higher than that in the same parts of the green varieties. For example, the anthocyanin content in leaves was 4 times, in stems was 3 times, and in flowers was 2.5 times higher in red varieties than in green varieties. The anthocyanin content in leaves was significantly higher than that in other organs. In summary, the anthocyanin content was significantly correlated with the color phenotype of *D. officinale*.

The relative expression levels of *DoMYB5* and *DobHLH24* in various parts of *D. officinale* were higher than those in green plants (Figure 1E), and they were clearly involved in the anthocyanin synthesis of *D. officinale*. For example, the relative expression of *DoMYB5* in flowers was 64 times that in red varieties and 7 times that in the leaves. The relative expression of *DobHLH24* in the stems of red varieties was 10 times that of green varieties (Figure 1F).

The anthocyanin content was significantly correlated with the expression of the two genes. The correlation coefficient between the anthocyanin content in different parts of red varieties and the corresponding *DoMYB5* expression was 0.85938, and the correlation coefficient with *DobHLH24* was slightly lower, namely, 0.51577. In green varieties, the above two values were 0.71216 and 0.749974, respectively. For the same gene, there was a significant correlation between its expression level and the expression site in different varieties. The correlation coefficient between the *DoMYB5* expression level and expression site was 0.96584, and that of *DobHLH24* was 0.94709.

### 2.2. Cloning and Characterization of DoMYB5 and DobHLH24

**Figure 2:**
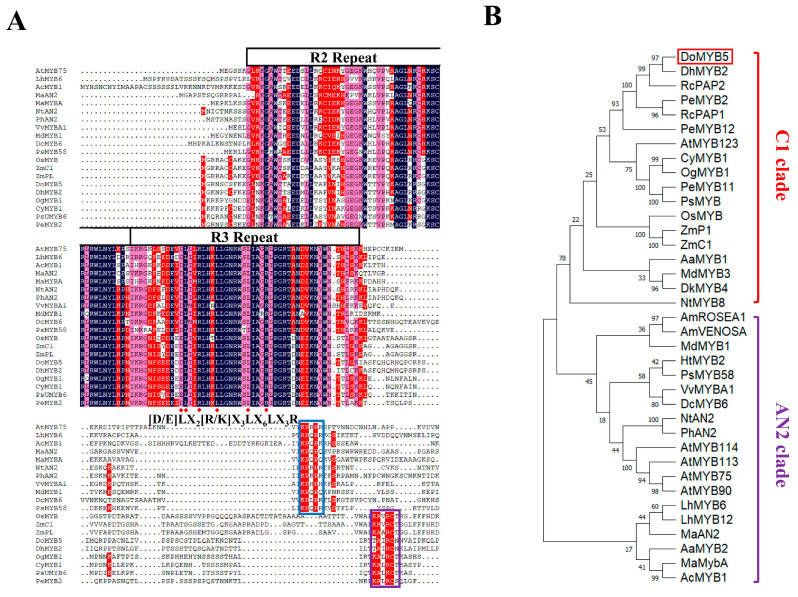

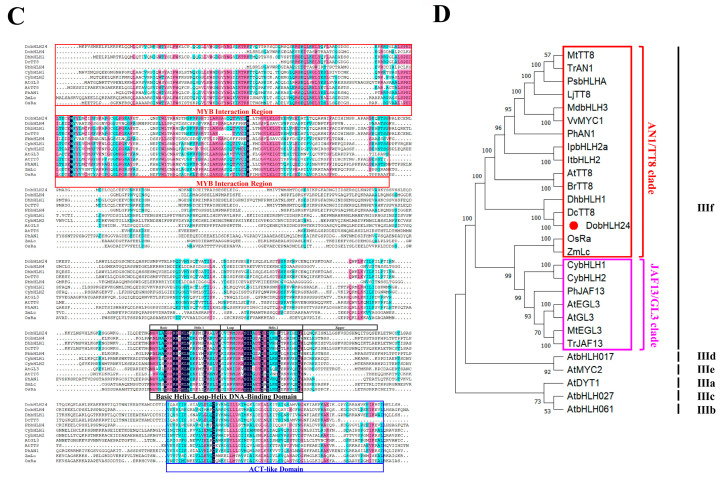
Sequence alignment and phylogenetic analysis of DoMYB5 and DobHLH24. (**A**,**C**) Alignment of the protein sequence of MYBs (**A**) and bHLHs (**C**) from different species. (**B**,**D**) Phylogenetic analysis of DoMYB5 (**B**) and DobHLH24 (**D**) with selected transcription factors from other plant species.

The full-length cDNA of *DoMYB5* was successfully obtained by PCR amplification. The ORF of *DoMYB5* was 873 bp, encoding 184 amino acids. Amino acid sequence analysis showed that DoMYB5 had a highly conserved R2R3 domain at the N-terminal. A protein core characteristic sequence interacting with the bHLH transcription factor, [D/E] LX2 [R/K] X3LX6LX3R, was found in the R3 domain of DoMYB5. A conserved motif related to anthocyanin regulation, KAX [K/R] C [S/T], was found in the C-terminal region of DoMYB5 (Figure 2A). Phylogenetic analysis showed that DoMYB5 and other MYBs that promote anthocyanin synthesis were grouped into one group (Figure 2B).

The ORF of *DobHLH24* was 1989 bp encoding 412 amino acids. There was a typical bHLH conservative region in the N-terminal region of the DobHLH24 protein, and a character sequence that can interact with MYB was also found in this region (Figure 2C). Phylogenetic analysis showed that the protein most closely related to DobHLH24 was DcTT8, which was also closely related to DhbHLH1 (Figure 2D).

### 2.3. Transient Overexpression of DoMYB5 and DobHLH24 in the Leaf of D. officinale

**Figure 3:**
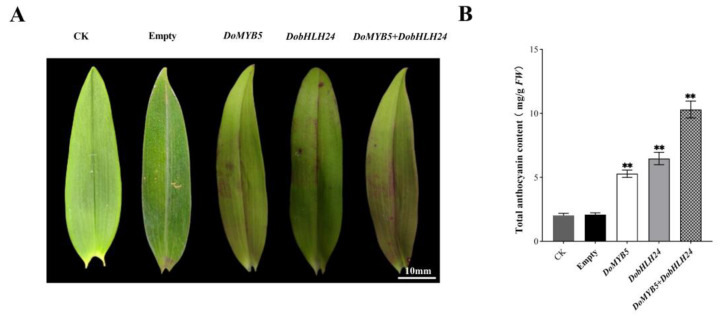
*DoMYB5* and *DobHLH24* regulated anthocyanin biosynthesis in *D. officinale*. (**A**) The phenotype of leaves transiently expressing empty vector, *DoMYB5*, *DobHLH24,* and *DoMYB5* + *DobHLH24* and the uninjected plant (CK). (**B**) Anthocyanin content was determined 10 days after injection into the leaf of *D. officinale*. ** Extremely significant difference (*p* < 0.01).

To further demonstrate the function of *DoMYB5* and *DobHLH24* in *D. officinale*, *A. tumefaciens* strain EHA105 harboring the recombinant plasmids of 35S::DoMYB5 and 35S::DobHLH24 were separately or simultaneously injected into the leaf of *D. officinale.* The overexpression of *DoMYB5* and *DobHLH24* in green varieties promoted the accumulation of anthocyanins. Significantly higher levels of anthocyanins were accumulated in the leaves overexpressing *DoMYB5* and *DobHLH24* compared with the control (Figure 3A,B). *DoMYB5* and *DobHLH24* were noticeably involved in regulating the accumulation of anthocyanins in *D. officinale*.

### 2.4. Stable Transformation of DoMYB5 and DobHLH24 in Tobacco

**Figure 4:**
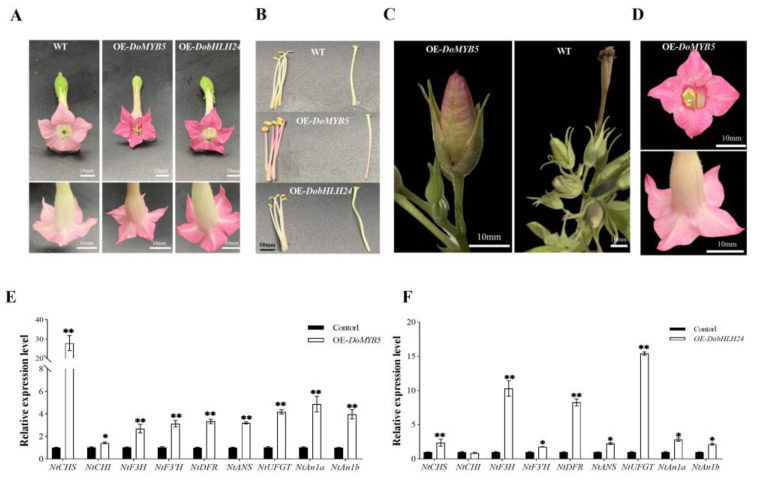
Phenotypic and relative expression levels of anthocyanin pathway genes of transgenic tobacco flowers overexpressing *DoMYB5* and *DobHLH24*. (**A**) Flowers of overexpressing *DoMYB5* and *DobHLH24* lines. (**B**) Stamens and pistil of overexpressing *DoMYB5* and *DobHLH24* lines. (**C**) Fruit of WT and overexpressing *DoMYB5* lines. (**D**) Overexpressing *DoMYB5* plants have a petal reduction phenotype. (**E**,**F**) Relative expression levels of anthocyanin pathway genes in flowers of transgenic tobacco lines carrying *DoMYB5*(E) and *DobHLH24*. (**F**). * Significant difference (*p* < 0.05). ** Extremely significant difference (*p* < 0.01).

The introduction of *DoMYB5* and *DobHLH24* genes deepened the color of the tobacco bud and fully developed corolla (Figure 4A). The stamens and pistils of *DoMYB5* transgenic plants turn red, while these tissues were green in wild-type plants (Figure 4B). The fruit of *DoMYB5* transgenic plants was dark red (Figure 4C). It is worth noting that the transgenic plants overexpressing *DoMYB5* temporarily showed significant changes in flower morphological characteristics. The wild-type tobacco plant had five petal flowers, but many transgenic lines overexpressing *DoMYB5* had four-petal flowers (Figure 4D). The expression of several key genes in the flavonoid biosynthesis pathway in the overexpression *DoMYB5* plant was significantly increased, and some of them were significantly higher than in the control group (Figure 4E). Except for *NtCHI*, all target genes detected in transgenic tobacco overexpressing *DobHLH24* were upregulated (Figure 4F). *NtAN1a* and *NtAN1b* were also detected, and they were upregulated in the *DoMYB5* transgenic lines. *DoMYB5* may regulate anthocyanin synthesis genes through interaction with endogenous *bHLH* in tobacco.

### 2.5. Verification of the Interaction between DobHLH24 and DoMYB5

**Figure 5:**
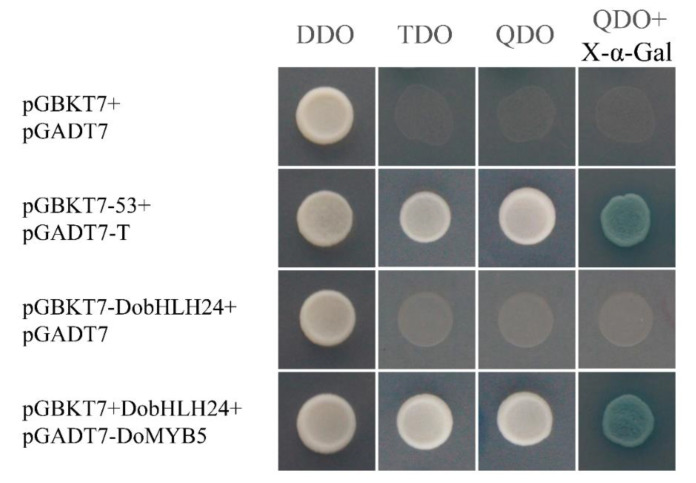
Analysis of the interaction between DobHLH24 and DoMYB5 in the Y2H experiments.

The results of Y2H showed that the four combinations could grow on a double dropout (DDO) SD medium. Only the positive control, the yeast co-transformed by DoMYB5 and DobHLH24, could grow on triple dropout (TDO) and quadruple dropout (QDO) SD media. Furthermore, the cell turned blue on the QDO SD medium with X-a-gal (Figure 5). This indicated that DoMYB5 and DobHLH24 could form heterodimers.

### 2.6. Regulation of DoMYB5 and DobHLH24 on the Promoters of DoCHS and DoDFR

**Figure 6:**
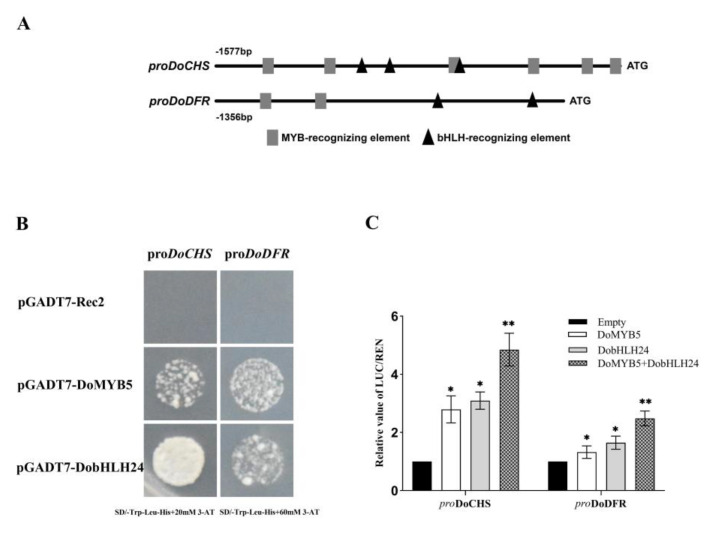
DoMYB5 and DobHLH24 activate the promoters of *DoCHS* and *DoDFR*. (**A**) Pattern diagram of the promoters of *DoCHS* and *DoDFR*. Triangles, bHLH-recognizing elements; rectangles, MYB-recognizing elements. (**B**) DoMYB5 and DobHLH24 bound directly to the promoters of *DoCHS* and *DoDFR* in yeast one-hybrid assays. (**C**) DoMYB5 and DobHLH24 activate the promoters of *DoCHS* and *DoDFR* in dual-luciferase assays. pro: promoter. * Significant difference (*p* < 0.05). ** Extremely significant difference (*p* < 0.01).

The promoters of *DoCHS* and *DoDFR* contain the MYB recognition element and the bHLH recognition element, respectively (Figure 6A). PHISDoCHS and pHISDFR were transformed into Y187 competent cells and cultured on SD selective medium (SD/-Trp-Leu+3-AT) containing 0–100 mM 3-AT. Yeast Y187 transformed with pHISDoCHS could be inhibited by 3-AT at 20 mM, while yeast Y187 transformed with pHISDFR could be inhibited by 3-AT at 60 mM. When DoMYB5 or DobHLH24 were co-expressed with *DoCHS* in Y187 yeast, yeast could grow on SD/-Trp-Leu-His medium containing 20 mM 3-AT, indicating that DoMYB5 and DobHLH24 could directly interact with the *DoCHS* promoter. Similarly, DoMYB5 and DobHLH24 could also directly interact with the *DoDFR* promoter (Figure 6B). The regulation of DoMYB5 and DobHLH24 on *DoCHS* and *DoDFR* was analyzed using a dual-luciferase assay. The results showed that both DoMYB5 and DobHLH24 could activate *DoCHS* and *DoDFR* promoters. Compared with acting alone, the co-expression of DoMYB5 and DobHLH24 could significantly improve the activity of *DoCHS* and *DoDFR* promoters, which was 4.8 times and 2.5 times higher, respectively, than the activity of the control group (Figure 6C).

## 3. Discussion

Anthocyanins are the main color-determining pigments [34,39,40]. MYB and bHLH are key transcription factors that regulate the biosynthetic pathway of plant anthocyanins [21,41,42,43,44,45]. In many plants, MYB, bHLH, and WD40 can regulate anthocyanin biosynthesis by forming MBW complexes [46,47,48,49,50]. In this paper, we cloned and analyzed an R2R3-MYB transcription factor DoMYB5 and a bHLH transcription factor DobHLH24 from *D. officinale*. The expression of the *DoMYB5* and *DobHLH24* in the red variety was significantly higher than that of the green variety, regardless of organs. These results suggest that they might play important roles in anthocyanin accumulation in *D. officinale*. The transient expression of *DoMYB5* and *DobHLH24* in *D. officinale* (Figure 3) and the stable transformation experiment in tobacco (Figure 4) both proved that *DoMYB5* and *DobHLH24* can promote the accumulation of anthocyanins. Similar results have been reported in other orchids. *PeMYB11* in *Phalaenopsis*, *RcPAP1* and *RcPAP2* in *Cattleya*, and *DhMYB2* and *DhbHLH1* in *Dendrobium* can promote the accumulation of anthocyanins [27,28,51]. At the same time, genes related to the anthocyanin pathway were upregulated in transgenic tobacco that was stably expressing. The expression of R2R3-MYB family members in tobacco, such as *StAN1-R1* [52], *AaMYB2* [53], and *LhMYB-12* [54], can regulate the biosynthesis of anthocyanins to varying degrees. Consequently, DoMYB5 and DobHLH24 are activators of anthocyanin synthesis in *D. officinale*.

According to the homology of amino acid sequences, the R2R3-MYB transcription factors involved in promoting anthocyanin synthesis are generally divided into two subgroups, namely, the AN2 subgroup and the C1 subgroup [55,56]. In most dicots, R2R3-MYBs regulating anthocyanin synthesis in flowers and fruits are in the AN2 subgroup [57]. R2R3-MYBs in different monocots belong to two subgroups (Figure 2B). For example, ZmC1 [58] in maize and PeMYB2, PeMYB11, PeMYB12, RcPAP1, RcPAP2, DoMYB5, and DhMYB2 in Orchidaceae belong to the C1 subgroup, with a typical conservative motif (KAX [K/R] C [S/T]) of monocots [19] (Figure 2A). MaAN2 and MaMybA [59] in *Muscari botryoides*, AcMYB1 [60] in onion, LhMYB6, LhMYB12 [61], and LvMYB52 [62] in lily belong to the AN2 subgroup. These R2R3-MYB contain the characteristic motif ([K/R] P [Q/R] P [Q/R] P [Q/R]) of the R2R3-MYBs associated with anthocyanin synthesis in dicots; however, they do not contain the conservative motif of the C1 subgroup of monocots (Figure 2A). Some monocots contain R2R3-MYBs of two subgroups at the same time, such as *Anthurium andraeanum*, which has both AN2 and C1 subgroups. AaMYB2 in the AN2 subgroup and AaMYB1, AaMYB4, and AaMYB5 in the C1 subgroup all play positive roles in anthocyanin synthesis [53,63]. The above-related studies indicate that although structural changes have occurred in R2R3-MYB in monocots, the function of promoting anthocyanin synthesis is nearly consistent.

MYB promotes anthocyanin synthesis by regulating key genes in the anthocyanin synthesis pathway [64]. In transgenic plants that were introduced into *PyMYB10* [65], *EsAN2* [66], and *SlAN2* [67], the expression levels of early and late genes for anthocyanin synthesis were increased. In our study, the expression levels of anthocyanin synthesis-related genes, namely, *CHS*, *CHI*, *F3H*, *F3′H*, *DFR*, *ANS*, and *DFGT*, in transgenic *DoMYB5* tobacco, were higher than those in wild-type plants (Figure 4). In Orchidaceae, MYB, which promotes anthocyanin synthesis, may control anthocyanin synthesis by regulating key genes at different stages of anthocyanin synthesis. *DhMYB2* [28], *PsUMYB6* [26], *CyMYB1* [31], and *ChMYB1* [68] can only promote late gene expression in the anthocyanin synthesis pathway, while *OgMYB1* [25], *PeMYB2*, and *PeMYB12* [27] can promote early and late gene expression.

In monocots, the mechanism of MYB regulating anthocyanin synthesis may have a low correlation with its amino acid sequence. There is no clear regularity in the mechanism of AN2 and C1 subgroups of R2R3-MYB transcription factors in anthocyanin synthesis in monocots. Both can activate the expression of EBGs or/and LBGs alone or in combination with bHLH. For example, in the AN2 subgroup, MaMybA and MaAN2 of hyacinth only regulate LBGs, but the former does not rely on the assistance of bHLH protein, while the latter must cooperate with bHLH [19,59]. LhMYB12 and LhMYB6 can interact with LhbHLH2 to regulate the expression of *LhCHSa* and *LhDFR*, respectively [54,69]. In the C1 subgroup, OgMYB1 of *Oncidium* can induce anthocyanin formation by simultaneously activating the expression of EBGs and LBGs, *OgCHI* and *OgDFR*, respectively [25]. ZmC1 can regulate the expression of related genes alone or in interaction with ZmLC [70]. The DhMYB2 of *Phalaenopsis* cannot bind to the *DhDFR* or *DhANS* promoters alone. The complex formed by DhMYB2 and DobHLH24 can only activate the expression of LBGs but cannot activate the expression of EBGs [28]. PeMYB11 in *Phalaenopsis* requires the assistance of PebHLH1. However, both the presence or absence of PebHLH1, PeMYB2, and PeMYB12 can activate the expression of *PeF3H5*, *PeDFR1*, and *PeANS3* [27]. In our study, DoMYB5 can bind directly to the promoters of *DoCHS* and *DoDFR* alone or form a complex with DobHLH24, activating EBGs and LBGs. At the same time, we also found that DoMYB5 and DhMYB2, both belonging to the C1 subgroup, are highly similar in terms of amino acid sequence, but their regulatory mechanisms are significantly different. A more precise examination of the R2R3-MYB transcription factors in *D. officinale* and other monocots should be necessary to determine how they promote the expression of anthocyanin synthesis genes.

## 4. Materials and Methods

### 4.1. Plant Materials

The two *D. officinale* varieties with different colors come from Guangdong Province and Fujian Province of China (Figure 1). The leaves, flowers, and stems of the varieties from Guangdong Province were noticeably red. The varieties from Fujian Province were green. Two samples of *D. officinale* were cultivated and preserved in the greenhouse of the Fujian Agriculture and Forestry University of China in March 2021. The two types of *D. officinale* used in the experiment were all biennial seedlings. Both *Nicotiana tabacum* (cv. K326) and *N. benthamiana* were cultivated in the conventional culture substrate for routine management. *N. tabacum* was used for subsequent gene transformation analysis, and *N. benthamiana* was used for dual-luciferase assays.

### 4.2. Flavonoid Extraction and Measurement

The extraction and content determination methods of anthocyanins were conducted according to Feng [71] with slight modifications. The fresh leaves of the third above-ground leaf position of green and red *D. officinale* varieties and their corresponding stem segments and intact petals were taken for anthocyanin content determination. After the samples were frozen in liquid nitrogen, they were ground into powder, then 0.5 g of each powder was added to 10 mL of a 2% hydrochloric acid/methanol solution. After ultrasonic extraction for 1 h, samples were centrifuged at 4 °C at 10,000 rpm for 10 min. The collected supernatants were the total anthocyanin extracts, and their absorption value at 530 nm was measured. Total anthocyanin content (mg/g FW) = A530 × MW × a × 1000/ε × 1, where A530 is the absorption value at 530 nm, MW is the molecular weight of anthocyanin-3-glucoside (449.38), a is the dilution multiple of the extract, and ε is the molar absorption coefficient of anthocyanin-3-glucoside (26,900). The anthocyanin content of each sample was expressed in mg equivalent of anthocyanin glycosides per gram of fresh weight. Each sample was extracted and measured three times.

### 4.3. DNA, RNA Isolation, and cDNA Synthesis

DNA samples were extracted using a DNAprep Pure Plant Kit (Tiangen Biochemical Technology Co., Ltd., Beijing, China) and used for promoter sequence clones. Total RNA was isolated from *D. officinale* and tobacco corolla limbs using the FastPure Plant Total RNA Isolation Kit (Polysaccharide- and Polyphenolic-rich) (Tiangen Biochemical Technology Co., Ltd., Beijing, China) following the manufacturer’s instructions. The quality of RNA and DNA samples was evaluated using 1% agarose gels and the purity was measured using NanoDrop (Thermo Scientific, Wilmington, DE, USA). RNA samples were converted to cDNA for RT-PCR analysis using a PrimeScript™ II 1st Strand cDNA Synthesis Kit (Takara Bio Inc., Otsu, Shiga, Japan) and for qRT-PCRanalysis with Hifair^®^ III 1st Strand cDNA Synthesis SuperMix (gDNA digester plus) (Yeasen Biotechnology Co., Ltd., Shanghai, China).

### 4.4. Isolation of the Full-Length cDNA of DoMYB5 and DobHLH24 from D. officinale

After analyzing the R2R3-MYB and bHLH transcription factor families of *D. officinale* based on a genome-wide perspective, combined with our group’s earlier transcriptome sequencing results and related reports [15,28], *DoMYB5* and *DobHLH24* were selected as the target genes for our focus of analysis in 2020. The cDNA sequences of *DoMYB5* and *DobHLH24* were obtained using conventional PCR techniques. Specific primers were designed by Primer 5.0 (Table S1). PCR amplification products were cloned into T/A cloning vector pMD18-T (Takara Bio Inc., Dalian, China) and sequenced for verification. The full-length amino acid sequences encoded by *DoMYB5* and *DobHLH24* were sequenced and phylogenetically analyzed using ClusterX2 and MEGA 11 programs (Neighbor-Joining method), respectively. The node support of the phylogenetic tree was assessed using 1000 bootstrap replicates. Sequences of R2R3-MYBs and bHLHs from other plants were obtained from GenBank (http://www.ncbi.nlm.nih.gov/, accessed on 20 March 2020). The MYB protein accessions were as follows: DoMYB5(XP_020672813), DhMYB2(KY039157), RcPAP2(MN420462), PeMYB2(AIS35919), RcPAP1(MN420461), PeMYB12(AIS35929), AtMYB123(Q9FJA2), CyMYB1(LC422758), OgMYB1(EF570115), PeMYB11(AIS35928), PsMYB(FJ039853), OsMYB(CAA75509), ZmP1(NP_001105885), ZmC1(NP_001106010), AmROSEA1(ABB83826), AmVENOSA(ABB83828), MdMYB1(ABK58138), HtMYB2(QJQ28877), PsMYB58(QZJ84669), VvMYBA1(BAD18977), DcMYB6(ARD08872), NtAN2(ACO52472), PhAN2(AAF66727), AtMYB114(Q9FNV8), AtMYB113(Q9FNV9), AtMYB75(NP_176057), AtMYB90(Q9ZTC3), LhMYB6(BAJ05399), LhMYB12(BAJ05398), MaAN2(ASF20090), AaMYB2(KU726561), MaMybA(AVD68967), AcMYB1(AQP25671), AaMYB1(AAO92352), MdMYB3(AEX08668), DkMYB4(AB503701), and NtMYB8(AUQ44163). The bHLHprotein accessions were as follows: MtTT8(KM892777), TrAN1(AIT76559), PsbHLHA(E3SXU4), LjTT8(BAH28881), MdbHLH3(ADL36597), VvMYC1(ACC68685), PhAN1(AAG25927), IpbHLH2a(ABW69686), ItbHLH2(BAD18984), AtTT8(Q9FT81), BrTT8(AEA03281), DhbHLH1(KY039158), DcTT8(PKU66073), DobHLH24(XM_020824205), OsRa(AAC49219), ZmLc(ABD72707), CybHLH1(LC422759), CybHLH2(LC422760), PhJAF13(AAC39455), AtEGL3(Q9CAD0), AtGL3(Q9FN69), MtEGL3(KEH21065), TrJAF13(AIT76563), AtbHLH017(AAM19778), AtMYC2(Q39204), AtDYT1(O81900), AtbHLH027(AAS79544), and AtbHLH061(AAM10950).

### 4.5. Transient Expression of DoMYB5 and DobHLH24 in D. officinale Leaves

*DoMYB5* and *DobHLH24* were inserted into the pTRV2e vector. The vectors used in the study were provided by the Horticultural Plant Genetics and Breeding Laboratory of Fujian Agricultural and Forestry University. The expression vector was constructed using a ClonExpress^®^ II One-Step Cloning Kit (Vazyme Biotech Co., Ltd., Nanjing, China). The vector pTRV2e-DoMYB5/DobHLH24 was constructed by inserting the DoMYB5/DobHLH24cDNA containing the open reading frame in the thepTRV2e plasmid. The details of primers used in vector construction are listed in Table S1. The constructed expression vectors were introduced into *Agrobacterium tumefaciens* EHA105. The experiment was carried out in the spring of 2021. The materials were taken from biennial plants of two types of *D. officinale*. We selected normal and 2-week-old healthy leaves of *D. officinale* as injection materials. Three kinds of engineered *A. tumefaciens* for transformation were obtained, including strains containing 35S::DoMYB5 or 35S::DobHLH24 alone, as well as a strain containing a mixture of the same amount of 35S::DoMYB5 and 35S::DobHLH24. The unmodified pTRV2e plasmid and uninjected plants were used as the control. The above strains were each transiently transformed into the leaves of *D. officinale*, and the changes in leaf color were observed after 10 days. The changes in leaf color and anthocyanin content in the corresponding injection area of the same injection leaf were recorded. The experiment was conducted with three biological replicates.

### 4.6. Stable Transformation of DoMYB5 and DobHLH24 in Tobacco

The expression vector used in the stable transformation was constructed by inserting *DoMYB5* and *DobHLH24* into the pSAK277 vector. The vector pSAK277-DoMYB5/DobHLH24 was constructed by inserting the DoMYB5/DobHLH24 cDNA containing the open reading frame in the pSAK277 plasmid. The details of primers used in vector construction are listed in Table S1. Approximately 6-week-old tobacco was transformed and regenerated according to Sparkes’ method [72]. The plant expression vector, pSAK277-DoMYB5/DobHLH24, was introduced into *Agrobacterium tumefaciens* GV3101. Tobacco leaf discs were infected with Agrobacterium tumefaciens for 15 min, washed with sterile water, and transferred to a bud induction medium containing 50 mg·L^−1^ kanamycin. Until regenerated adventitious buds were obtained, they were transferred to the root induction medium. The fully blooming flowers of T0 tobacco plants were collected for subsequent analysis.

### 4.7. Real-Time Quantitative PCR

The qRT-PCR analysis was completed with Hieff^®^ qPCR SYBR Green Master Mix (No Rox) (Yeasen Biotechnology Co., Ltd., Shanghai, China). The details of primers are listed in Table S1. The expression levels of *DoMYB5* and *DobHLH24* in the flowers, leaves, and stems of green and red *D. officinale* varieties were determined by fluorescent quantitative PCR, with *DoACTIN* serving as an internal reference gene. The key genes of the anthocyanin pathway in stable transformation materials of tobacco were determined, and *NtACTIN* was used as the internal reference gene. The expression level of each gene was calculated using the 2^−ΔΔCT^ method [73]. The experiment was conducted with three biological replicates.

### 4.8. Yeast One-Hybrid (Y1H) Assay

Specific primers were designed according to the promoter sequences of *DoCHS* and *DoDFR* (Table S1). The promoter sequences of these two genes were cloned by nested PCR. The cis-acting elements of the two promoters were analyzed using the online software PlantPAN 3.0 (http://plantpan.itps.ncku.edu.tw/index.html, accessed on 10 August 2021). For the yeast one-hybrid assay, the promoters of *DoCHS* and *DoDFR* were inserted into the pHIS2 vector, and *DoMYB5* and *DobHLH24* were inserted into the pGADT7-Rec2 vector. The primers used in vector construction are listed in Table S1. The constructed recombinant plasmid vectors pGADT7-Rec2 and pHIS2 were each transformed into the Y187 yeast strain. Then, 3-amino-1,2,4-triazole (3-AT), at different concentrations, was added to the synthetic defined (SD)-Trp/-His medium as the selective medium. The growth of yeast was observed after 5 days of culture at 30 °C. The appropriate concentration of 3-AT was determined by observing the growth of yeast in different media. The constructed recombinant plasmid vectors pGADT7-Rec2 and pHIS2 were co-transformed into the Y187yeast strain and cultured in SD/-Trp-Leu and SD/-Trp-Leu-His medium. The growth of yeast was observed after 3 days of culture at 30 °C. The pGADT7-Rec2 vector was used as the control.

### 4.9. Yeast Two-Hybrid (Y2H) Assay

For yeast two-hybrid analysis, the *DoMYB5* gene was connected to the pGBKT7 vector, and the *DobHLH24* gene was connected to the pGADT7 vector using the infusion method (Table S1). Y2H was analyzed according to Nakatsuka et al. [74]. After detecting the toxicity and self-activation ability of the bait protein of pGBKT7-DobHLH24, pGBKT7-DobHLH24, and pGBKT7-DoMYB5 were co-transformed into Y2H Gold yeast cells. Then, the transformed yeast cells were cultivated in order in SD/-Trp-Leu, SD/-Trp-Leu-His, SD/-Trp-Leu-His-Ade, and SD/-Trp-Leu-His-Ade+X-ɑ-Gal medium. The growth of yeast was observed after 3 days of culture at 30 °C. The pGBKT7 and pGADT7 vectors were used as negative controls, and the pGBKT7-53 and pGADT7-T vectors were used as positive controls.

### 4.10. Dual Luciferase Assay

For dual-luciferase analysis, the promoters of *DoCHS* and *DoDFR* were separately introduced into the vector pGreenII0800, and the *DoMYB5* and *DobHLH24* were separately introduced into the vector pSAK277 using the infusion method (Table S1). Two vectors from different combinations were co-injected into tobacco leaves. The tobacco leaves co-injected by pGreenII0800-DoCHS-LUC and pGreenII0800-DoDFR-LUC were used as the control. At 48 h after injection, tobacco leaf samples were collected, and the enzyme activities of firefly luciferase (LUC) and sea kidney luciferase (REN) were measured using a dual-luciferase reporter assay kit (Vazyme Biotech Co., Ltd., Nanjing, China) with reference to Yin’s method [75]. All of the above tests were set with no less than three biological replicates.

## 5. Conclusions

Two MYB and bHLH transcription factors, namely, *DoMYB5* and *DhbHLH24*, were cloned from *D. officinale*. The expression level of *DoMYB5* and *DhbHLH24* were positively correlated with the plant anthocyanin content. The transient expression in the leaves of *D. officinale* and the stable expression in tobacco significantly promoted the accumulation of anthocyanins. Both can regulate the promoters of *DoCHS* and *DoDFR* and increase the expression level of the latter. The effect of combined regulation was obviously stronger than that of single regulation. The results provide clues to reveal the mechanism of MYB and bHLH in the color determination of *D. officinale* and provide a new idea for cultivating *D. officinale* varieties with high anthocyanin contents.

## Data Availability

Not applicable.

## Supplementary Materials

The following supporting information can be downloaded at: https://www.mdpi.com/article/10.3390/ijms24087552/s1. Table S1: Primers used in this study.
