# Supplementary material for: DoMYB5 and DobHLH24, Transcription Factors Involved in Regulating Anthocyanin Accumulation in Dendrobium officinale
Source: Int J Mol Sci. 2023 Apr 20;24(8):7552. doi: 10.3390/ijms24087552 (PMC10142772; doi:10.3390/ijms24087552)
Supplement: Supplementary file 1 [file ijms-24-07552-s001.zip › ijms-2294040-supplementary.pdf]

Table S1. Primers used in this study.

Primer Name	Sequence
**For PCR**	
DoMYB5-F	5′-ATGGGAAGGAATTCGTGCAG-3′
DoMYB5-R	5′-CTAGAATCCACCTCCAATATCAAATAAAG-3′
DobHLH24-F	5′-ATGAAACCAGTCAGCATGGAAGAG-3′
DobHLH24-R	5′-TTAATGGGATAAGAGTTCATGAATTGTCC-3′
proDoCHS-1F	5′-ACTTCCACGGGACCACTACTTGAGGC-3′
proDoCHS-1R	5′-GCTGGTTGATGGATGGAAGCTTGAGAAG-3′
proDoCHS-2F	5′-ACTTCCACCGGACCACTACC-3′
proDoCHS-2R	5′-GCTGGTTGATGGATGGAAGCTT-3′
proDoDFR-1F	5′-TTCAACTCACTCTCTAGTCTTATC-3′
proDoDFR-1R	5′-GAATATTTCAGTCTCAGTTTATTAAAGA-3′
**For qPCR in *Dendrobium officinale***	
q*DoMYB5*-F	5′-TAATCCGAACAACGGCAATAAG-3′
q*DoMYB5*-R	5′-AGCATCCAGGTCTCAAAATCC-3′
q*DobHLH24*-F	5′-CGTCGCAAAGCGAGAGATTG-3′
q*DobHLH24*-R	5′-TTGTTCTGCGCTTCTTTGCC-3′
q*DoActin*-F	5′-TCCCAAGGCAAACAGAGAAA-3′
q*DoActin*-R	5′-GGCCACTAGCATATAGGGAAAG-3′
**Transient *Dendrobium officinale* transformation**	
DoMYB5-pTRV2e-F	5′-CGACGACAAGACCCTGCAG<br>ATGGGAAGGAATTCGTGCAG-3′
DoMYB5-pTRV2e-R	5′-GAGGAGAAGAGCCCTGCAG<br>CTAGAATCCACCTCCAATATCA-3′
DobHLH24-pTRV2e-F	5′-CGACGACAAGACCCTGCAG<br>ATGAAACCAGTCAGCATGGAAGA-3′
DobHLH24-pTRV2e- R	5′-GAGGAGAAGAGCCCTGCAG<br>TTAATGGGATAAGAGTTCATGAATT-3′
**Transient tobacco transformation**	
DoMYB5-pSAK277-F	5′-ACTAGTGGATCCAAAGAATTC<br>ATGGGAAGGAATTCGTGCAGTTT-3′
DoMYB5-pSAK277-R	5′-GAAGTACTCTCGAGAAGCTT<br>CTAGAATCCACCTCCAATATCAAAT-3′
DobHLH24-pSAK277-F	5′-ACTAGTGGATCCAAAGAATTC<br>ATGAAACCAGTCAGCATGGA-3′
DobHLH24-pSAK277-R	5′-GAAGTACTCTCGAGAAGCTT<br>TTAATGGGATAAGAGTTCAT-3′
**For qPCR in transgenic tobacco**	
q*NtCHS*-F	5′-GTACAACTAGTGGTGTAGACA-3′
q*NtCHS*-R	5′-CCAACTTCACGAAGGTGAC-3′
q*NtCHI*-F	5′-GAAATCCTCCGATCCAGTGA-3′
q*NtCHI*-R	5′-CAACGTTGACAACATCAGGC-3′
q*NtF3H*-F	5′-ACAGGGTGAAGTGGTCCAAG-3′

q*NtF3H*-R	5′-CCTTGGTTAAGGCCTCCTTC-3′
q*NtF3'H*-F	5′-TCCAAGAATACTGGCCCAAG-3′
q*NtF3'H*-R	5′-CTCACAACTCTCGGATGCAA-3′
q*NtDFR*-F	5′-AACCAACAGTCAGGGGAATG-3′
q*NtDFR*-R	5′-TTGGGCATCGAGAGTTCCAG-3′
q*NtANS*-F	5′-TGGCGTTGAAGCTCATACTG-3′
q*NtANS*-R	5′-GGAATTAGGCACACACTTTGC-3′
q*NtUFGT*-F	5′-CAATGTTTGGGATGGTGTCA-3′
q*NtUFGT*-R	5′-TTCCTCCTCTGCCTCTTTCA-3′
q*NtAn1a*-F	5′-ACCATTCTCGAACACCGAAG-3′
q*NtAn1a*-R	5′-TGCTAGGGCACAATGTGAAG-3′
q*NtAn1b*-F	5′-CTTGAACACTTCTCAAACCGA-3′
q*NtAn1b*-R	5′-TGCTAGGGCACAATGTGAAG-3′
q*NtActin*-F	5′-AATGATCGGAATGGAAGCTG-3′
q*NtActin*-R	5′-TGGTACCACCACTGAGGACA-3′
**For yeast one-hybrid analysis**	
DoMYB5-pGADT7-Rec2-F	5′-ACCAGATTACGCTCATATG ATGGGAAGGAATTCGTGCAGTT-3′
DoMYB5-pGADT7-Rec2-R	5′-CGATTCATCTGCAGCTCGAG CTAGAATCCACCTCCAATATCA-3′
DobHLH24-pGADT7-Rec2-F	5′-ACCAGATTACGCTCATATG ATGAAACCAGTCAGCATGGAAGA-3′
DobHLH24-pGADT7-Rec2-R	5′-CGATTCATCTGCAGCTCGAG TTAATGGGATAAGAGTTCATGAATT -3′
proDoCHS-pHIS2-F	5′-CTATAGGGCGAATTC ACTTCCACCGGACCACTACCT-3′
proDoCHS-pHIS2-R	5′-GCGAACGCGTGAGCTC GCTGGTTGATGGATGGAAGCTT-3′
proDoDFR-pHIS2-F	5′-CTATAGGGCGAATTC TTCAACTCACTCTCTAGTCTTATCA -3′
proDoDFR-pHIS2-R	5′-GCGAACGCGTGAGCTC GAATATTTCAGTCTCAGTTTATTAA -3′
**For yeast two-hybrid analysis**	
DoMYB5-AD-F	5′-ACCAGATTACGCTCATATG ATGGGAAGGAATTCGTGCAGTT-3′
DoMYB5-AD-R	5′-CGATTCATCTGCAGCTCGAG CTAGAATCCACCTCCAATATCA-3′
DobHLH24-BD-F	5′-TCAGAGGAGGACCTGCATATG ATGAAACCAGTCAGCAT-3′
DobHLH24-BD-R	5′-TTCGGCCTCCATGGCCATATG TTAATGGGATAAGAGTTC-3′
**Dual luciferase assay**	
DoMYB5-pSAK277-F	5′-ACTAGTGGATCCAAAGAATTC ATGGGAAGGAATTCGTGCAGTTT-3′

DoMYB5-pSAK277-R	5′-GAAGTACTCTCGAGAAGCTT CTAGAATCCACCTCCAATATCAAAT-3′
DobHLH24-pSAK277-F	5′-ACTAGTGGATCCAAAGAATTC ATGAAACCAGTCAGCATGGA-3′
DobHLH24-pSAK277-R	5′-GAAGTACTCTCGAGAAGCTT TTAATGGGATAAGAGTTCAT-3′
proDoCHS- pGreenII0800-F	5′-ACTATAGGGCGAATTGGGTACC ACTTCCACCGGACCACTACCT -3′
proDoCHS- pGreenII0800-R	5′-TGCAGGAATTCGATATCAAGCTT GCTGGTTGATGGATGGAAGCTT -3′
proDoDFR- pGreenII0800-F	5′-TCGACGGTATCGATAAGCTT TTCAACTCACTCTCTAGTCTTATCA-3′
proDoDFR- pGreenII0800-R	5′-GCTCTAGAACTAGTGGATCC GAATATTTCAGTCTCAGTTTATTAA-3′
